# Exploring the Association Between Platelet Count, the Systemic Immune Inflammation Index, and Fracture Risk in Postmenopausal Women with Osteoporosis: A Cross-Sectional Study

**DOI:** 10.3390/jcm14155453

**Published:** 2025-08-02

**Authors:** Cecilia Oliveri, Anastasia Xourafa, Rita Maria Agostino, Valentina Corigliano, Antonino Botindari, Agostino Gaudio, Nunziata Morabito, Alessandro Allegra, Antonino Catalano

**Affiliations:** 1 Department of Clinical and Experimental Medicine, University Hospital of Messina, 98124 Messina, Italy; 2 Unit of Talassemia, University Hospital of Catania, 95123 Catania, Italy; 3 Unit of Oncology, Grand Metropolitan Hospital “Bianchi Melacrino Morelli”, 89128 Reggio Calabria, Italy; 4 Department of Clinical and Experimental Medicine, University Hospital of Catania, 95123 Catania, Italy; agostino.gaudio@gmail.com; 5 Division of Hematology, Department of Human Pathology in Adulthood and Childhood “Gaetano Barresi”, University Hospital of Messina, 98124 Messina, Italy

**Keywords:** platelets, systemic immune inflammation index, blood count, fracture risk, bone mineral density, vitamin D, osteoporosis, older adults

## Abstract

**Background/Objectives:** Platelets play a role in bone metabolism and fracture healing. This study aimed to investigate the association between platelet indices and the derived systemic immune inflammation index (SII) with fracture risk in postmenopausal women. **Methods:** Platelet count, mean platelet volume, platelet distribution width (PDW), platelet crit, percentage of large platelets (P-LCR), platelet–lymphocyte ratio, and the SII, calculated as (NxP)/L, where N, P, and L represented neutrophils, platelets and lymphocytes counts, respectively, were evaluated. Bone mineral density (BMD) was measured using dual-energy X-ray absorptiometry. **Results:** A total of 124 women (mean age 68.4 ± 9 years) were stratified into two groups based on the median platelet count; the “lower platelet count group” (*n* = 58) had a count of 200,000 (174,000 to 226,000), while the “higher platelet count group” (*n* = 66) had a count of 281,500 (256,500 to 308,500). The higher platelet count group showed a higher hip fracture risk (7.4 vs. 4.5%, *p* = 0.08) and lower lumbar spine BMD (0.773 vs. 0.83 gr/cm^2^, *p* = 0.03). By dividing the participants into two groups with higher SSI (950,848.6 ± 746,097.99) (*n* = 61) and lower SII (355,751.2 ± 88,662.6) (*n* = 63), the group with the higher SII showed the higher hip fracture risk (7.4 vs. 3.6%, *p* = 0.01). Univariate regression analysis revealed correlations between chronological age and PDW (r = 0.188, *p* = 0.047), and P-LCR (r = 0.208, *p* = 0.03), as well as associations between vitamin D status and P-LCR (r = −0.301, *p* = 0.034), and between SII and hip fracture risk (r = 0.12, *p* = 0.007). **Conclusions:** Platelet count and SII were associated with fracture risk in postmenopausal women undergoing osteoporosis assessment. Given their reproducibility and cost-effectiveness, these markers warrant further investigation in future prospective studies focused on bone fragility.

## 1. Introduction

Osteoporosis is a systemic condition marked by reduced bone mass, deterioration of both the micro- and macro-architecture of the skeleton, and qualitative changes that increase susceptibility to fractures. It is among the leading causes of morbidity and mortality in the elderly population worldwide [1]. Several studies have demonstrated a strong association between osteoporosis and inflammatory disorders, including chronic inflammatory bowel disease, rheumatoid arthritis, systemic lupus erythematosus (SLE), and chronic obstructive pulmonary disease (COPD) [2].

Beyond overt inflammatory diseases, physiological states such as menopause and aging are also characterized by “low-grade inflammation,” defined by persistent, low-level inflammatory activity. These processes are well known to correlate with a decline in bone mineral density (BMD). In postmenopausal women, declining estrogen levels promote the production of proinflammatory cytokines such as tumor necrosis factor-alpha (TNF-α) and interleukin-17 (IL-17), which in turn stimulate osteoblasts to increase expression of Receptor Activator of Nuclear Factor Kappa-B Ligand (RANKL), thereby accelerating bone remodeling [3]. Aging is associated with elevated circulating cytokines, contributing to systemic inflammation and exerting widespread effects that, in addition to osteoporosis, play a role in the development of sarcopenia, cardiovascular disease, and renal dysfunction. Inflammatory activity is particularly prominent during fracture healing [4], with cytokine expression peaking shortly after the fracture, declining during cartilage formation, and rising again during the remodeling phase [5].

Although platelets are traditionally associated with hemostasis, they also play crucial immunomodulatory roles that influence both innate and adaptive immunity [6]. Platelet granules contain cytokines such as IL-1 and substantial amounts of transforming growth factor-beta (TGF-β), which, depending on the local cytokine milieu, may drive differentiation toward either anti-inflammatory regulatory T cells (Tregs) or proinflammatory Th17 cells [7]. Platelets interact with neutrophils and monocytes via molecules such as P-selectin and CD40, forming platelet–leukocyte aggregates that modulate innate immune responses [8]. Emerging evidence suggests that platelets also contribute to bone remodeling. Following a fracture, activated platelets aggregate and release growth factors including platelet-derived growth factor (PDGF), vascular endothelial growth factor (VEGF), insulin-like growth factor 1 (IGF-1), epidermal growth factor (EGF), and TGF-β, which recruit osteogenic cells to the injury site [9]. Platelet count has previously been linked to osteoporosis in certain studies [10]. Investigations into platelet indices such as mean platelet volume (MPV) and platelet distribution width (PDW) have yielded inconsistent findings. Some studies suggest that elevated MPV and PDW are associated with lower BMD [11,12], while others report reduced MPV and PDW in individuals with osteoporosis [13]. A higher platelet count, even within the normal range, is frequently observed in individuals with insulin resistance, obesity, metabolic syndrome, and cardiovascular disease [14,15]. Elevated PDW is also seen in hematological disorders such as myeloproliferative syndromes, indicating that platelet metrics may reflect broader pathophysiological conditions beyond bone health [16,17].

In recent years, increasing attention has been directed toward the use of inflammatory indices derived from complete blood counts—such as the neutrophil-to-lymphocyte ratio (NLR), platelet-to-lymphocyte ratio (PLR), and the systemic immune inflammation index (SII)—as indirect markers of chronic inflammation [18]. These indices, obtainable from routine blood tests, provide an integrated view of immune status: an increase in neutrophils or platelets (key components of innate immunity and thrombosis), coupled with a relative decrease in lymphocytes (central to adaptive immunity), may indicate systemic inflammation [19]. Although traditionally studied in cardiovascular, metabolic, and oncologic contexts—where elevated NLR and PLR values correlate with poorer outcomes, increased mortality, and higher complication rates—these indices have recently gained attention in bone and mineral metabolism research as well [20].

Several studies have reported an inverse relationship between NLR or PLR and BMD, showing that individuals with osteopenia or osteoporosis tend to have higher neutrophil-to-lymphocyte or platelet-to-lymphocyte ratios compared to healthy controls [21,22,23,24,25]. These findings support the concept of “immunoporosis,” which posits that chronic immune activation exacerbates bone loss by promoting osteoclastogenesis. Among emerging markers, the systemic immune inflammation index (SII) has emerged as a potentially comprehensive snapshot of inflammatory status, combining neutrophil, platelet, and lymphocyte counts into a single measure (neutrophils × platelets/lymphocytes) [26]. Initially explored in oncology, preliminary studies have suggested a possible link between elevated SII values and postmenopausal osteoporosis, implying that a persistently heightened inflammatory environment may accelerate bone loss [27,28].

The aim of our study was to investigate the association of platelet count and SII with fracture risk in a setting of postmenopausal women.

## 2. Materials and Methods

Postmenopausal women referred to the outpatient clinics for bone and mineral disorders at the University Hospital of Messina, Italy, were consecutively enrolled. Eligibility criteria included a history of spontaneous menopause and a diagnosis of osteoporosis or osteopenia, as defined by the World Health Organization (WHO) criteria. Osteoporosis was diagnosed based on T-score values ≤ −2.5 standard deviations (SD), while osteopenia was defined by T-scores between −1 and −2.5 SD. Both clinical fragility fractures and morphometric vertebral fractures—identified via spinal X-rays as prescribed by specialists—were considered in the assessment. Exclusion criteria included the presence of thyroid disorders (hypo- or hyperthyroidism), parathyroid diseases (e.g., primary hyperparathyroidism or hypoparathyroidism), a known history of malignancy, heart failure, renal failure, respiratory failure, or liver failure. All participants provided written informed consent for the use of their data for research purposes. The study protocol, part of a broader research project, was approved by the local Ethics Committee (Protocol No. 71/19) and conducted in accordance with the Declaration of Helsinki and its subsequent amendments.

Fracture risk was calculated using the FRAX^®^ algorithm (https://frax.shef.ac.uk/FRAX/tool.aspx?lang=it, accessed on 10 April 2025), which estimates the 10 yr probability of major osteoporotic fractures and hip fractures. The FRAX tool integrates multiple clinical risk factors, including age, gender, body weight, height, personal history of fractures, parental history of hip fractures, alcohol consumption, smoking status, corticosteroid use, rheumatoid arthritis and secondary osteoporosis diagnosis. To facilitate further correlation analysis, femoral neck BMD was not included in the FRAX calculation.

Areal BMD was measured at the lumbar spine and proximal femur using dual-energy X-ray absorptiometry (DXA), the gold-standard technique, in anteroposterior projection. Measurements were performed using a Hologic Discovery Wi densitometer - 35 Crosby Drive, Bedford, MA 01730 USA - which was calibrated daily and had a coefficient of variation of 0.5%. BMD values were expressed in g/cm^2^ and as T-scores (SD), as previously described [29,30].

The following hematological parameters were analyzed: platelet count, mean platelet volume (MPV)—indicative of platelet production rates, platelet distribution width (PDW)—reflecting platelet anisocytosis, plateletcrit (PCT)—representing total platelet mass per unit of blood volume, platelet Large Cell Coefficient (P-LCC), Platelet Large Cell Ratio (P-LCR), white blood cell count, leukocyte differential, and inflammatory indices, including neutrophil-to-lymphocyte ratio (NLR), platelet-to-lymphocyte ratio (PLR), and the systemic immune inflammation index (SII), calculated as (neutrophils × platelets)/lymphocytes. Blood samples were obtained at 8:00 a.m. after an overnight fast and abstinence from smoking. Complete blood counts were performed immediately using an automated cell counter. Serum 25(OH)D was measured by high performance liquid chromatography—mass spectrometry. All laboratory analyses were conducted at the University Hospital of Messina.

**Figure 1:**
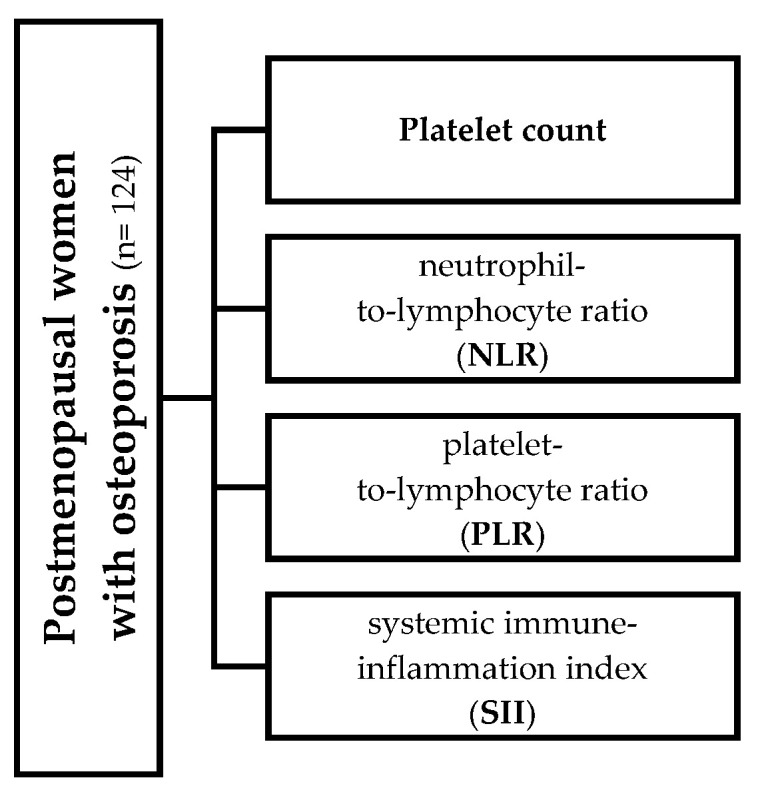
Variables for all of which (in relation to their median value) the population was stratified into two groups.

Participants were stratified into two groups based on the median values of platelet count and the aforementioned inflammatory indices (Figure 1). Comparative analyses were then conducted between these groups. Statistical analyses were performed using MedCalc software (version 10.2.0.0). The Kolmogorov–Smirnov test was used to assess the normal distribution of the data. Values were reported as mean ± SD or median (interquartile range). Spearman’s univariate regression analysis served to assess associations between the studied variables. Group comparisons were conducted using Student’s *t*-test or the Mann–Whitney U test, depending on data distribution. A *p*-value of < 0.05 was considered statistically significant for all the analyses.

## 3. Results

The main clinical characteristics of the study population are summarized in Table 1.

**Figure 2:**
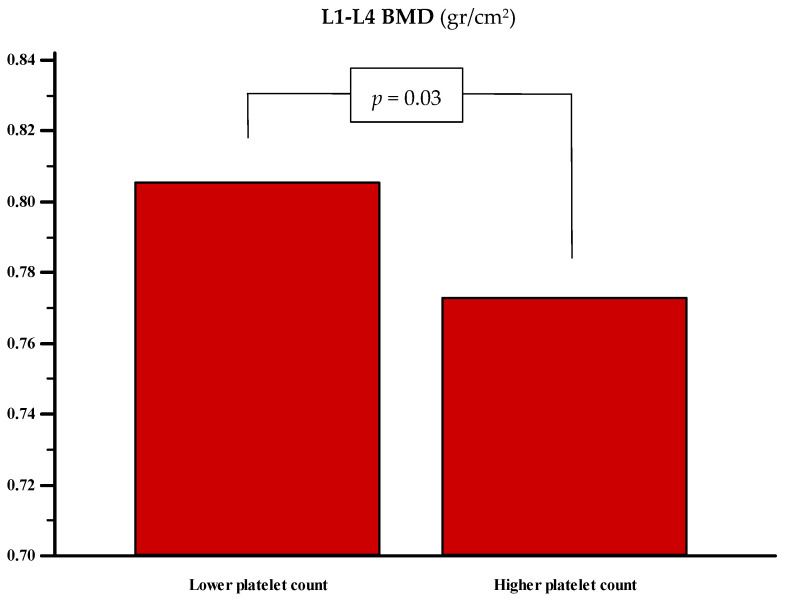
Bone mineral density at the lumbar spine in women with lower [200,000 (174,000 to 226,000)] (*n* = 58) and higher [281,500 (256,500 to 308,500)] platelet counts (*n* = 66). The statistical significance was assessed with the Mann–Whitney U test.

A total of 124 postmenopausal women were enrolled. The population was initially stratified based on the median platelet count, resulting in two groups: a lower platelet count group (below the median, *n* = 58) and a higher platelet count group (above the median, *n* = 66). The median platelet count was 200,000/μL (IQR: 174,000–226,000) in the lower group and 281,500/μL (IQR: 256,500–308,500) in the higher group. No significant differences were observed between the two groups in terms of age, BMI, vitamin D status, or 10-yr probability of major osteoporotic fractures. However, a borderline significant difference was noted in the 10 yr probability of hip fracture, which was higher in the group with the elevated platelet count (7.4% vs. 4.5%, *p* = 0.08). Additionally, lumbar spine BMD was significantly lower in the higher platelet count group (0.773 vs. 0.83 g/cm^2^, *p* = 0.03) (see Figure 2).

**Figure 3:**
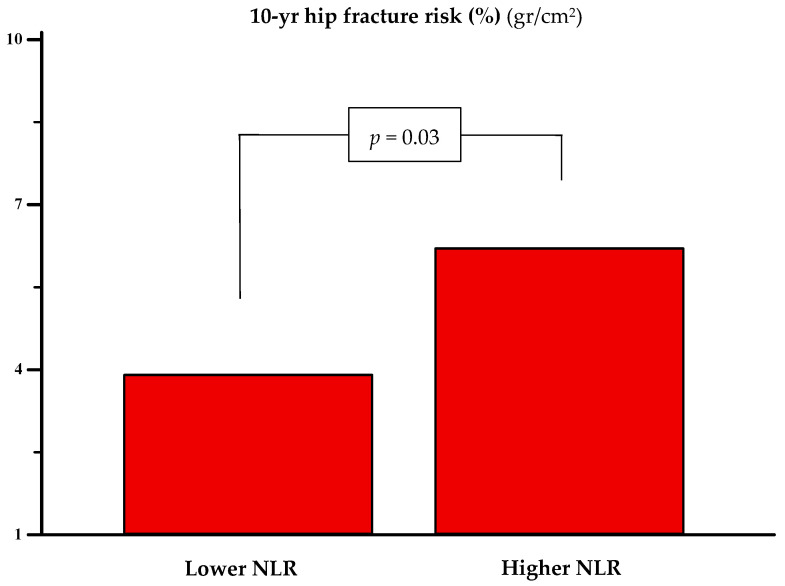
The 10 yr risk of hip fracture in women with lower (*n* = 64) [1.58 (1.32 to 1.78)] and higher (*n* = 60) [3 (2.37 to 3.75)] neutrophil-to-lymphocyte ratio (NLR) values. The statistical significance was assessed with the Mann–Whitney U test.

When participants were stratified by the neutrophil-to-lymphocyte ratio (NLR) into higher (*n* = 60) and lower (*n* = 64) NLR groups—median values of 3.00 (IQR: 2.37–3.75) vs. 1.58 (IQR: 1.32–1.78), respectively—only the 10 yr hip fracture risk differed significantly. The group with higher NLR had a greater estimated risk (6.2% vs. 3.9%, *p* = 0.03) (see Figure 3).

In contrast, no significant differences in BMD (lumbar spine or femoral neck) or osteoporotic fracture risk were observed when the population was divided by the platelet-to-lymphocyte ratio (PLR). The higher PLR group (*n* = 61) had a median value of 165.9 (IQR: 150.1–211.6), while the lower PLR group (*n* = 63) had a median of 105.9 (IQR: 91.1–121.3).

**Figure 4:**
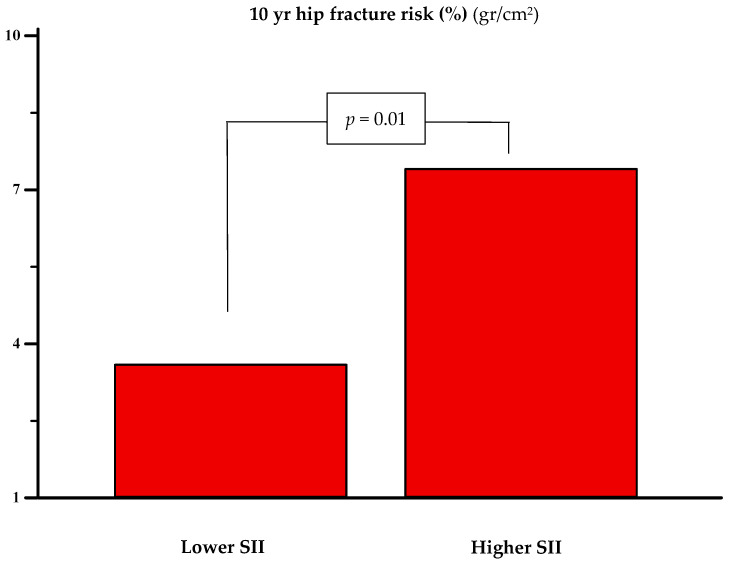
**The** 10 yr risk of hip fracture in women with lower (*n* = 63) [720,923 (600,348.2 to 1,055,226.3)] and higher (*n* = 61) [351,807.1 (292,745.2 to 430,781.2)] systemic immune inflammation index (SII). The statistical significance was assessed with the Mann–Whitney U test.

Finally, when stratified by the systemic immune inflammation index (SII) into higher (*n* = 61) and lower (*n* = 63) groups—720,923 (IQR: 600,348.2–1,055,226.3) vs. 351,807.1 (IQR: 292,745.2–430,781.2), respectively—a significant difference in 10 yr hip fracture risk was observed. The higher SII group had a significantly greater risk (7.4% vs. 3.6%, *p* = 0.01) (see Figure 4).

Univariate regression analysis revealed the following significant associations: chronological age was positively associated with PDW (r = 0.188, *p* = 0.047) and P-LCR (r = 0.208, *p* = 0.03); the 10 yr hip fracture risk was positively associated with NLR (r = 0.3, *p* = 0.02) and SII (r = 0.35, *p* = 0.007); the lumbar spine BMD was negatively associated with platelet count (r = −0.284, *p* = 0.003) and showed a trend with PCT (r = −0.206, *p* = 0.074); vitamin D status was negatively associated with P-LCC (r = −0.301, *p* = 0.034); NLR was positively associated with PLT count (r = 0,02; *p* = 0.35).

## 4. Discussion

This study demonstrates that platelet count and the systemic immune inflammation index (SII) are valuable markers for assessing fracture risk in postmenopausal women with osteoporosis. Given the often asymptomatic nature of osteoporosis, early detection is frequently reliant on the identification of associated risk factors—some of which may influence fracture risk independently of bone mineral density (BMD). Despite growing awareness of bone health, the social and economic burden of chronic diseases, including osteoporosis, is projected to increase in the coming years. Therefore, early identification of individuals at high fracture risk remains a clinical priority.

In this context, several hematological and inflammatory biomarkers have been proposed to assist clinicians in screening and risk stratification. Prior studies have highlighted associations between BMD and parameters derived from routine blood tests, such as hemoglobin concentration, red blood cell distribution width, platelet count, and composite indices like NLR, PLR, and SII [18,19,20,21,22,23,24,25,26,27,28,31,32]. Notably, SII has recently emerged as a potential predictor of fragility fractures in postmenopausal women, capable of distinguishing between individuals at high and low fracture risk [27]. Similarly, elevated NLR has been linked to a higher incidence of vertebral and femoral neck fractures [33].

These findings are consistent with the pathophysiological role of inflammation in bone loss, as supported by inverse correlations between BMD and inflammatory markers such as CRP, IL-1, IL-6, and TNF-α [34,35,36]. Chronic inflammatory diseases—such as rheumatoid arthritis, inflammatory bowel disease, and COPD—frequently coexist with bone and muscle mass loss [2,3]. This phenomenon is also observed in physiological states of low-grade inflammation, including menopause and aging, where declining estrogen levels promote the release of pro-osteoclastogenic cytokines (e.g., TNF-α, IL-17, IL-6), activating the RANK/RANKL/OPG pathway [3,34,35,36]. The resulting imbalance in bone remodeling, favoring resorption over formation, is central to osteoporosis pathogenesis. Within this framework, NLR and PLR offer clinical insight into systemic inflammation. Elevated NLR reflects increased neutrophil counts, often associated with heightened proinflammatory cytokine activity, while high PLR underscores the immunomodulatory role of platelets. Our findings support this model: women with higher platelet counts, even within the normal range, exhibited significantly increased hip fracture risk and lower lumbar spine BMD. Moreover, a positive association between NLR and platelet count was detected, and both NLR and SII were significantly associated with increased hip fracture risk, reinforcing their potential utility as surrogate markers of inflammation-related bone fragility.

An inverse relationship between vitamin D status and both PLR and NLR has been previously reported, suggesting that chronic inflammation and vitamin D deficiency may synergistically impair bone metabolism [37]. In our study, chronological age was positively correlated with PDW and P-LCR, indicating increased platelet heterogeneity and reactivity with aging—possibly due to inflammaging or compensatory changes in megakaryocyte function. These findings align with evidence linking aging to a prothrombotic state and increased risk of cardiovascular, oncologic, and neurodegenerative diseases [38]. Furthermore, the inverse association between vitamin D and P-LCC suggests a regulatory role of vitamin D on platelet activation and size. Since P-LCC quantifies large, reactive platelets rich in growth factors like PDGF and VEGF, this relationship may reflect vitamin D’s immunomodulatory effects on megakaryocyte maturation and systemic inflammation. In vitro studies support this hypothesis, showing that vitamin D influences platelet-derived growth factor release, potentially contributing to bone repair [37,38]. These findings are consistent with clinical data supporting vitamin D’s role in bone preservation [7,39] and immune modulation [40,41,42,43,44,45].

Our results are also in line with previous studies in populations with diabetes [46,47], further emphasizing the link between fragility fracture risk, BMD, and inflammatory biomarkers. The associations observed with NLR, SII, and platelet count suggest that routine blood count parameters—often available from unrelated clinical evaluations—could support opportunistic screening for osteoporosis.

Moreover, the well-established relationship between fracture risk and cardiovascular risk in postmenopausal women deserves attention. Patients with osteoporosis may exhibit signs of vascular aging, reflecting shared pathophysiological mechanisms between bone and cardiovascular diseases [48,49,50]. Recent studies have shown an inverse correlation between BMD and the 10 yr ASCVD risk score, suggesting that bone loss may serve as a marker of cardiovascular risk [51]. Additionally, correlations between coagulation biomarkers, bone turnover markers, and BMD have been reported in older adults [52], supporting the hypothesis that platelet count and SII may reflect both cardiovascular and skeletal fragility, particularly in the context of postmenopausal inflammation.

This study has several limitations that should be acknowledged. The relatively small sample size and cross-sectional design limit the ability to draw causal inferences. Additionally, the analysis did not account for potential confounding factors such as skin pigmentation, physical activity levels, comorbidities (e.g., hypertension or diabetes mellitus), or socio-economic status. The lack of direct measurements of key inflammatory biomarkers (e.g., C-reactive protein, interleukin-1, interleukin-6, tumor necrosis factor-alpha) further constrains mechanistic interpretations. Nonetheless, a notable strength of the study lies in the homogeneity of the sample, which comprised exclusively postmenopausal women, thereby enhancing the internal validity of the findings.

Prospective longitudinal studies are needed to further elucidate the relationship between inflammatory biomarkers and fracture risk, and to determine whether targeted modulation of these markers can effectively reduce the incidence of fragility fractures.

## 5. Conclusions

This study identified a significant association between fracture risk and both platelet count and inflammatory markers—specifically the neutrophil-to-lymphocyte ratio (NLR) and the Systemic immune inflammation index (SII)—derived from routine blood counts in a cohort of postmenopausal women with osteoporosis. Given the cost-effectiveness and widespread availability of complete blood count testing, these findings suggest that such parameters could enhance clinicians’ ability to predict fracture risk in everyday practice.

Moreover, these indices may serve as valuable inputs for future artificial intelligence models aimed at improving risk stratification and guiding preventive strategies. However, to validate and generalize these findings, longitudinal studies are urgently needed. Such research should aim to confirm the predictive value of these markers over time and explore their integration into clinical decision-making tools for osteoporosis management.

## Figures and Tables

**Table 1:** Main characteristics of the participants.

	Total Participants (*n* = 124)
Age (yr)	68.4 ± 9
Menopausal age (yr)	49.8 ± 2.4
BMI (kg/m^2^)	24.8 ± 5.8
Bone mineral density	
L1-L4 BMD (gr/cm^2^)	0.8 (0.77 to 0.82)
Femur neck BMD (gr/cm^2^)	0.62 (0.6 to 0.62)
Osteoporosis [*n* (%)]	60 (48.4)
Osteopenia [*n* (%)]	64 (51.6)
10-yr probability of fracture	
Major osteoporotic (%)	16 (14 to 18)
Hip fracture (%)	5.4 (2.25 to 6.6)
Platelet count and indices	
Platelet count	239,000 (230,000 to 251,016)
Mean Platelet Volume (MPV) (fL)	10.2 (9.92 to 10.40)
Platelet Distribution Width (PDW) (%)	16.1 (16.00 to 16.20)
Plateletcrit (PCT) (%)	0.246 (0.23 to 0.26)
Platelet Large Cell Coefficient (P-LCC) (mm^2^)	62,500 (59,000 to 73,652.35)
Platelet Large Cell Ratio (P-LCR) (%)	27.35 (25.33 to 28.76)
Red blood cells (mm^2^)	4511.921 ± 495.914
White blood cells (mm^2^)	7.241 ± 3.190
Neutrophils (%)	62 ± 9.7
Lymphocytes (%)	29.8 ± 9.3
Inflammation markers	
Neutrophil-to-Lymphocyte Ratio (NLR)	2.1 (1.875 to 2.250)
Platelet-to-Lymphocyte Ratio (PLR)	132.12 (123.98 to 144.26)
Systemic immune inflammation index (SII)	48.997 (45.473 to 55.480)
Vitamin D status	
25(OH)D (ng/mL)	42.3 ± 15.5

Data are expressed as mean ± SD or median (IQR).

## Data Availability

The original contributions presented in this study are included in the article. Further inquiries can be directed to the corresponding authors.

## References

[1] Lamichhane A.P. (2005). Osteoporosis-an update. JNMA J. Nepal. Med. Assoc..

[2] Baker-LePain J.C., Nakamura M.C., Lane N.E. (2011). Effects of inflammation on bone: An update. Curr. Opin. Rheumatol..

[3] Delmas P.D. (2008). Clinical potential of RANKL inhibition for the management of postmenopausal osteoporosis and other metabolic bone diseases. J. Clin. Densitom..

[4] Mazzaferro S., De Martini N., Rotondi S., Tartaglione L., Ureña-Torres P., Bover J., Pasquali M., ERA-EDTA Working Group on CKD-MBD (2020). Bone, inflammation and chronic kidney disease. Clin. Chim. Acta..

[5] Giannoudis P.V., Hak D., Sanders D., Donohoe E., Tosounidis T., Bahney C. (2015). Inflammation, bone healing, and anti-inflammatory drugs: An update. J. Orthop. Trauma..

[6] Semple J.W., Italiano J.E., Freedman J. (2011). Platelets and the immune continuum. Nat. Rev. Immunol..

[7] Silvagno F., De Vivo E., Attanasio A., Gallo V., Mazzucco G., Pescarmona G. (2010). Mitochondrial localization of vitamin D receptor in human platelets and differentiated megakaryocytes. PLoS ONE.

[8] Salari Sharif P., Abdollahi M. (2010). The role of platelets in bone remodeling. Inflamm. Allergy Drug Targets.

[9] Kark L.R., Karp J.M., Davies J.E. (2006). Platelet releasate increases the proliferation and migration of bone marrow-derived cells cultured under osteogenic conditions. Clin. Oral Implants Res..

[10] Kim J., Kim H.S., Lee H.S., Kwon Y.J. (2020). The relationship between platelet count and bone mineral density: Results from two independent population-based studies. Arch. Osteoporos..

[11] Li X.S., Zhang J.R., Meng S.Y., Li Y., Wang R.T. (2012). Mean platelet volume is negatively associated with bone mineral density in postmenopausal women. J. Bone Miner. Metab..

[12] Yan P., Xu Y., Wan Q., Feng J., Yang J., Li H., Zhong H., Gao C., Zhang Z. (2018). Impact of MPV and PDW on bone mineral density and their relationship with osteoporosis in Chinese patients with type 2 diabetes. Int. J. Clin. Exp. Med..

[13] Akbal A., Gokmen F., Gencer M., Inceer B.S., Komurcu E. (2014). Mean platelet volume and platelet distribution width can be related to bone mineralization. Osteoporos. Int..

[14] Ly H.Q., Kirtane A.J., Murphy S.A., Buros J., Cannon C.P., Braunwald E., Gibson C.M. (2006). Association of platelet counts on presentation and clinical outcomes in ST-elevation myocardial infarction (from the TIMI trials). Am. J. Cardiol..

[15] Samocha-Bonet D., Justo D., Rogowski O., Saar N., Abu-Abeid S., Shenkerman G., Shapira I., Berliner S., Tomer A. (2008). Platelet counts and platelet activation markers in obese subjects. Mediat. Inflamm..

[16] Osselaer J.C., Jamart J., Scheiff J.M. (1997). Platelet distribution width for differential diagnosis of thrombocytosis. Clin. Chem..

[17] Kim J.Y., Yoon J., Lim C.S., Choi B.M., Yoon S.Y. (2015). Clinical significance of platelet-associated hematological parameters as an early supplementary diagnostic tool for sepsis in thrombocytopenic very-low-birth-weight infants. Platelets.

[18] Tamhane U.U., Aneja S., Montgomery D., Rogers E.K., Eagle K.A., Gurm H.S. (2008). Association between admission neutrophil to lymphocyte ratio and outcomes in patients with acute coronary syndrome. Am. J. Cardiol..

[19] Dong C.H., Wang Z.M., Chen S.Y. (2018). Neutrophil to lymphocyte ratio predict mortality and major adverse cardiac events in acute coronary syndrome: A systematic review and meta-analysis. Clin. Biochem..

[20] Templeton A.J., McNamara M.G., Šeruga B., Vera-Badillo F.E., Aneja P., Ocaña A., Leibowitz-Amit R., Sonpavde G., Knox J.J., Tran B. (2014). Prognostic role of neutrophil-to-lymphocyte ratio in solid tumors: A systematic review and meta-analysis. J. Natl. Cancer Inst..

[21] Özturk Z.A., Yesil Y., Kuyumcu M.E., Bilici M., Özturk N., Yesil N.K., Özkaya M., Kısacık B., Kepekçi Y., Arıoğul S. (2013). Inverse relationship between neutrophil lymphocyte ratio (NLR) and bone mineral density (BMD) in elderly people. Arch. Gerontol. Geriatr..

[22] Yilmaz H., Uyfun M., Yilmaz T.S., Namuslu M., Inan O., Taskin A., Cakmak M., Bilgic M.A., Bavbek N., Akcay A. (2014). Neutrophil-lymphocyte ratio may be superior to C-reactive protein for predicting the occurrence of postmenopausal osteoporosis. Endocr. Regul..

[23] Huang C., Li S. (2016). Association of blood neutrophil lymphocyte ratio in the patients with postmenopausal osteoporosis. Pak. J. Med. Sci..

[24] Lee S.H., Ryu S.Y., Park J., Shin M.H., Han M.A., Choi S.W. (2019). The relationship of neutrophil-lymphocyte ratio and platelet-lymphocyte ratio with bone mineral density in Korean postmenopausal women. Chonnam Med. J..

[25] Eroglu S., Karatas G. (2019). Platelet/lymphocyte ratio is an independent predictor for osteoporosis. Saudi Med. J..

[26] Koseoglu S.B. (2017). Bone loss & platelet-to-lymphocyte ratio. Biomark. Med..

[27] Fang H., Zhang H., Wang Z., Zhou Z., Li Y., Lu L. (2020). Systemic immune-inflammation index acts as a novel diagnostic biomarker for postmenopausal osteoporosis and could predict the risk of osteoporotic fracture. J. Clin. Lab. Anal..

[28] Du Y.N., Chen Y.J., Zhang H.Y., Wang X., Zhang Z.F. (2021). Inverse association between systemic immune-inflammation index and bone mineral density in postmenopausal women. Gynecol. Endocrinol..

[29] Catalano A., Oliveri C., Natale G., Agostino R.M., Squadrito G., Gaudio A., Gembillo G., Marina D., Cernaro V., Longhitano E. (2024). Renal function is associated with changes in bone mineral density in postmenopausal osteoporotic women treated with denosumab: Data from a retrospective cohort study. J. Clin. Med..

[30] Oliveri C., Xourafa A., Morabito N., Di Giovanni A., Lupo E., Basile G., Gaudio A., Catalano A. (2025). Calf circumference predicts changes of bone mineral density in postmenopausal osteoporotic women receiving denosumab. Aging Clin. Exp. Res..

[31] Cesari M., Pahor M., Lauretani F., Penninx B.W., Bartali B., Russo R., Cherubini A., Woodman R., Bandinelli S., Guralnik J.M. (2005). Bone density and hemoglobin levels in older persons: Results from the InCHIANTI study. Osteoporos. Int..

[32] Pepe J., Colangelo L., De Martino V., Occhiuto M., Iervolino D., Pasqualetti P., Minisola S., Cipriani C. (2023). Study of the link between hematopoietic and skeletal systems in patients attending a referral center for osteoporosis. J. Endocrinol. Investig..

[33] Zhu H., Li Z., Zhou Y., Zheng R., Diao C., Li K., Feng Q., Wang D. (2022). Neutrophil-lymphocyte ratio as a risk factor for osteoporotic vertebrae fractures and femoral neck fractures. Medicine.

[34] De Pablo P., Cooper M.S., Buckley C.D. (2012). Association between bone mineral density and C-reactive protein in a large population-based sample. Arthritis Rheum..

[35] Wu Z.J., He J.L., Wei R.Q., Liu B., Lin X., Guan J., Lan Y.B. (2015). Creactive protein and risk of fracture: A systematic review and doseresponse meta-analysis of prospective cohort studies. Osteoporos. Int..

[36] Lin C.C., Li T.C., Liu C.S., Yang C.W., Lin C.H., Hsiao J.H., Meng N.H., Lin W.Y., Liao L.N., Li C.I. (2016). Associations of TNF-alpha and IL-6 polymorphisms with osteoporosis through joint effects and interactions with LEPR gene in Taiwan: Taichung Community Health Study for Elders (TCHS-E). Mol. Biol. Rep..

[37] Akbas E.M., Gungor A., Ozcicek A., Akbas N., Askin S., Polat M. (2016). Vitamin D and inflammation: Evaluation with neutrophil-to-lymphocyte ratio and platelet-to-lymphocyte ratio. Arch. Med. Sci..

[38] Elizondo-Montemayor L., Castillo E.C., Rodríguez-López C., Villarreal-Calderón J.R., Gómez-Carmona M., Tenorio-Martínez S., Nieblas B., García-Rivas G. (2017). Seasonal Variation in Vitamin D in Association with Age, Inflammatory Cytokines, Anthropometric Parameters, and Lifestyle Factors in Older Adults. Mediat. Inflamm..

[39] Bellone F., Catalano A., Sottile A.R., Gaudio A., Loddo S., Corica F., Morabito N. (2021). Early Changes of VEGF Levels After Zoledronic Acid in Women With Postmenopausal Osteoporosis: A Potential Role of Vitamin D. Front. Med..

[40] Atteritano M., Mirarchi L., Venanzi-Rullo E., Santoro D., Iaria C., Catalano A., Lasco A., Arcoraci V., Lo Gullo A., Bitto A. (2018). Vitamin D Status and the Relationship with Bone Fragility Fractures in HIV-Infected Patients: A Case Control Study. Int. J. Mol. Sci..

[41] Zhang P., Xu Q., Zhu R. (2024). Vitamin D and allergic diseases. Front. Immunol..

[42] Oteri G., Cicciù M., Peditto M., Catalano A., Loddo S., Pisano M., Lasco A. (2016). Does Vitamin D3 Have an Impact on Clinical and Biochemical Parameters Related to Third Molar Surgery. J. Craniofac. Surg..

[43] Hamza F.N., Daher S., Fakhoury H.M.A., Grant W.B., Kvietys P.R., Al-Kattan K. (2023). Immunomodulatory Properties of Vitamin D in the Intestinal and Respiratory Systems. Nutrients.

[44] Lauretani F., Salvi M., Zucchini I., Testa C., Cattabiani C., Arisi A., Maggio M. (2023). Relationship between Vitamin D and Immunity in Older People with COVID-19. Int. J. Environ. Res. Public Health.

[45] De Martinis M., Allegra A., Sirufo M.M., Tonacci A., Pioggia G., Raggiunti M., Ginaldi L., Gangemi S. (2021). Vitamin D Deficiency, Osteoporosis and Effect on Autoimmune Diseases and Hematopoiesis: A Review. Int. J. Mol. Sci..

[46] Xue Y., Bao W., Huang W., Zou X., Guo Y. (2024). Relationship between neutrophil-to-lymphocyte ratio, monocyte-to-lymphocyte ratio, platelet-to-lymphocyte ratio and osteoporosis in postmenopausal type 2 diabetic patients: A retrospective study. Medicine.

[47] Yin W., Li X., Zheng S., Lai W., Chen C., He X., Gong K., He K., Hu S., Zheng J. (2025). Association of novel inflammatory markers with osteoporosis index in older spine osteoporosis patients: NHANES 1999-2018 cross-sectional study. Sci. Rep..

[48] Gaudio A., Xourafa A., Zanoli L., Rapisarda R., Catalano A., Signorelli S.S., Castellino P. (2020). Early vascular ageing biomarkers in osteoporotic outpatients: A pilot study. Sci. Rep..

[49] Sharafi M.H., Nazari A., Cheraghi M., Souri F., Bakhshesh M. (2025). The link between osteoporosis and cardiovascular diseases: A review of shared mechanisms, risk factors, and therapeutic approaches. Osteoporos. Int..

[50] Gaudio A., Fiore V., Rapisarda R., Sidoti M.H., Xourafa A., Catalano A., Tringali G., Zanoli L., Signorelli S.S., Fiore C.E. (2017). Sclerostin is a possible candidate marker of arterial stiffness: Results from a cohort study in Catania. Mol. Med. Rep..

[51] Wani K., Sabico S., Veronese N., Al-Masri A.A., Al-Daghri N.M. (2025). Ten-year atherosclerotic cardiovascular disease risk score in post-menopausal women with low bone mineral density. Aging Clin. Exp. Res..

[52] Wang Z., Wang R., Ge H., Gu Y., Xian S., Yan L., Du G., Shen Z., Lv S., Zhan H. (2024). The correlation between coagulation biomarkers, bone turnover markers, and bone mineral density in Chinese adults aged ≥ 50 years. Arch. Med. Sci..

